# Natural Killer Cells in the Lungs

**DOI:** 10.3389/fimmu.2019.01416

**Published:** 2019-06-25

**Authors:** Jingjing Cong, Haiming Wei

**Affiliations:** ^1^Hefei National Laboratory for Physical Sciences at Microscale, CAS Key Laboratory of Innate Immunity and Chronic Disease, Division of Molecular Medicine, School of Life Sciences, University of Science and Technology of China, Hefei, China; ^2^Institue of Immunology, University of Science and Technology of China, Hefei, China; ^3^Division of Life Science and Medicine, The First Affiliated Hospital of USTC, University of Science and Technology of China, Hefei, China

**Keywords:** natural killer cells, lung, homeostasis, inflammation, infection, lung cancer

## Abstract

The lungs, a special site that is frequently challenged by tumors, pathogens and other environmental insults, are populated by large numbers of innate immune cells. Among these, natural killer (NK) cells are gaining increasing attention. Recent studies have revealed that NK cells are heterogeneous populations consisting of distinct subpopulations with diverse characteristics, some of which are determined by their local tissue microenvironment. Most current information about NK cells comes from studies of NK cells from the peripheral blood of humans and NK cells from the spleen and bone marrow of mice. However, the functions and phenotypes of lung NK cells differ from those of NK cells in other tissues. Here, we provide an overview of human and mouse lung NK cells in the context of homeostasis, pathogenic infections, asthma, chronic obstructive pulmonary disease (COPD) and lung cancer, mainly focusing on their phenotype, function, frequency, and their potential role in pathogenesis or immune defense. A comprehensive understanding of the biology of NK cells in the lungs will aid the development of NK cell-based immunotherapies for the treatment of lung diseases.

## Introduction

The lungs comprise mucosae that are constantly exposed to environmental and autologous stimuli, and they are sites of high incidence of primary and metastatic tumors (1). Accordingly, a rapid and efficient immune response that prevents tumorigenesis and pathogen invasion without leading to excessive inflammation is needed to maintain pulmonary homeostasis. As a type of innate immune cell, natural killer (NK) cells are regarded as the host's first line of defense against tumors and viral infection (2). Moreover, involvement of NK cells in various lung diseases, such as lung cancer, chronic obstructive pulmonary disease (COPD) and asthma, as well as infections, has been documented (Table 1) (34, 35, 37–39).

**Table 1:** Beneficial and/or detrimental roles of NK cells in mouse models of pulmonary disorders.

**Pathology**	**Beneficial role of NK cells**	**Detrimental role of NK cells**	**References**
**VIRUSES**			
*Influenza virus*	Promote host defense via IFN-γ at medium-dose	Induce immunopathology at high-dose	(3–6)
*Respiratory syncytial virus*	Inhibit type 2 inflammation via IFN-γ; promote host defense via IFN-γ	Exacerbate early acute lung injury via IFN-γ	(7–10)
*Herpes simplex virus*	Promote host defense via IFN-γ and cytotoxicity		(11, 12)
**BACTERIA**			
*Klebsiella pneumoniae*	Promote host defense via IL-22 and IFN-γ		(13, 14)
*Streptococcus pneumoniae*	Promote early clearance of bacteria in WT mice (3 h post infection)	Amplify pulmonary and systemic inflammation in scid mice; impair clearance of bacteria in scid mice (24 h post infection)	(15, 16)
*Pseudomonas aeruginosa*	Promote host defense via NKG2D and IFN-γ		(17, 18)
*Mycobacterium tuberculosis*	Promote host defense via IFN-γ in T cell-deficient mice		(19)
*Bordetella pertussis*	Promote host defense via IFN-γ		(20)
*Staphylococcus aureus*	Promote host defense via IFN-γ and TNF		(21, 22)
*Haemophilus influenzae*	Promote host defense via IFN-γ		(23)
*Chlamydia trachomatis*	Promote host defense via regulation of Th1/Treg and Th17/Treg balances		(24)
**FUNGI**			
*Aspergillus fumigatus*	Promote host defense via IFN-γ		(25, 26)
*Cryptococcus neoformans*	Promote host defense via IFN-γ		(27, 28)
Asthma	Promote inflammation resolution via clearance of eosinophils and CD4^+^ T cells in OVA-induced asthma	Promote allergic sensitization via initiation of type 2 response in OVA-induced asthma; promote pathogenesis via NKG2D and granzyme B in HDM-induced asthma?	(29–33)
COPD		Kill autologous lung epithelial cells	(34)
Lung cancer	Inhibit tumorigenesis in *Kras*-driven cancer; inhibit lung metastasis of cancer cells		(35, 36)

In humans, NK cells are usually defined as CD3^−^CD56^+^ cells, and they are divided into two main subsets with different functions and maturation statuses: CD56^bright^CD16^−^ and CD56^dim^CD16^+^. The CD56^dim^CD16^+^ NK cells are known as a highly differentiated subset with killer cell immunoglobulin-like receptor (KIR) expression, potent cytotoxicity and the capacity to induce antibody-dependent cellular cytotoxicity (ADCC), while the less mature CD56^bright^CD16^−^ NK cells lack KIR expression but are the major producers of cytokines (40–42). In mice, NK cells do not express CD56 and have historically been defined as CD3^−^NK1.1^+^ cells (and, more recently, CD3^−^NKp46^+^ cells). However, type 1 innate lymphoid cells (ILC1s) and some subsets of type 3 innate lymphoid cells (ILC3s) also express NK1.1 and NKp46 and are easily confused with NK cells (43, 44). No equivalent subsets to the human NK cell subsets have been established to date among mouse NK cells. Mouse NK cells are divided into four subsets from the most immature to the most mature, according to the expression of CD27 and CD11b: CD27^−^CD11b^−^, CD27^+^CD11b^−^, CD27^+^CD11b^+^, and CD27^−^CD11b^+^ (45– 47).

NK cell functions are modulated by the balance between activating and inhibitory signals delivered by receptors expressed on the NK cell surface (Table 2). Abnormal cells (including cancer cells and infected cells) activate NK cells via lack of ligands of NK cell inhibitory receptors (missing self) or increased expression of ligands of NK cell activating receptors (induced self) (48–50). In addition, cytokines such as interleukin (IL)-12, IL-15, IL-18, and type I interferon (IFN), as well as Toll-like receptor (TLR) ligands, are powerful activators of NK cell functions (51, 52). Activated NK cells then function in various environments mainly through cytotoxicity and cytokine production. Recent findings have revealed that the functions and phenotypes of NK cells vary depending on their local microenvironments (53), mainly due to the distinct cytokines, cellular composition and foreign stimuli of various tissues. In this review, we provide an overview of the current understanding and gaps in knowledge regarding NK cells in the lungs.

**Table 2:** Main surface markers of the lung NK cells discussed in this review.

**Relevance and function**	**NK cell-surface molecules**
Activating receptors	NKG2D, DNAM1^h^, NKp30^h^, NKp44^h^, NKp46, NKp80^h^, CD16
Inhibitory receptors	CD94/NKG2A, ILT2^h^, KIR2DL^h^, KIR2DL2^h^
Activation marker	CD69
Mature and differentiation markers	KIR^h^, CD57^h^,CD11b, CD43, CD49b, CD122, Ly49s^m^
Tissue-resident markers	CD49a, CD69, CD103
Adhesion molecules	CD11b, CD49a, CD49b, CD57^h^, CD103,

h *Expressed only by human NK cells*.

m *Expressed only by mouse NK cells*.

## Lung NK Cells in Homeostasis

**Figure 1:**
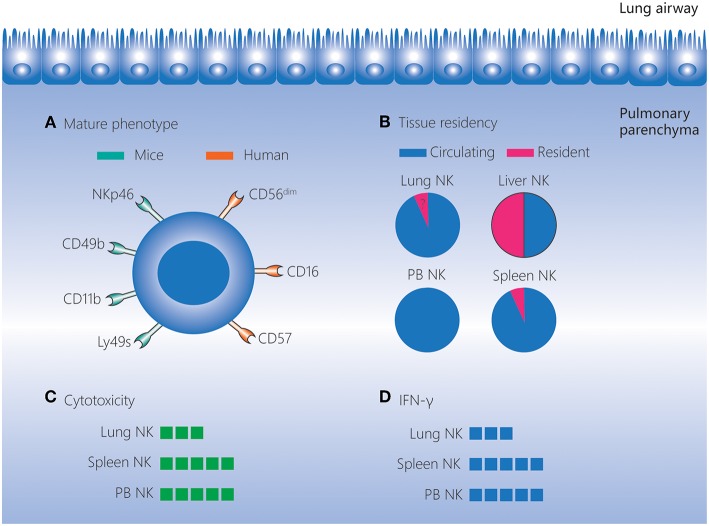
Lung NK cells in homeostasis. NK cells account for 10–20% of lymphocytes in the human and mouse lungs, and these cells are located in the lung parenchyma. **(A)** NK cells in the lungs have a more mature phenotype compared to those in other tissues. In mice, lung NK cells express high levels of the mature markers NKp46, CD49b, CD11b, and Ly49s. In humans, lung NK cells are mostly composed of the CD56^dim^CD16^+^ subset, and highly differentiated CD57^+^NKG2A^−^ NK cells are present at a higher frequency in the lungs than in matched peripheral blood. **(B)** The vast majority of lung NK cells are circulating, and the existence of a small percentage of seemingly tissue-resident NK cells in the lungs remains to be confirmed. **(C,D)** Lung NK cells are hypofunctional in homeostasis, and their cytotoxicity and IFN-γ production levels are lower than those of NK cells in the spleen and peripheral blood. IFN, interferon; NK, natural killer; PB, peripheral blood.

Lung NK cells are generally thought to originate and develop in the bone marrow, and then migrate to the lungs (54). In human lungs, NK cells, accounting for about 10–20% of the lymphocytes, are located in the parenchyma and are not detected outside the parenchyma (Figure 1) (1). In mice, lung NK cells account for about 10% of the lymphocytes, and this percentage is higher than the percentages in other tissues (liver, peripheral blood, spleen, bone marrow, thymus and lymph node) (55, 56). Moreover, the number of mouse lung NK cells is second only to the number of spleen NK cells (55).

Human lung NK cells are mostly composed of the CD56^dim^CD16^+^ subset. In addition, KIR-expressing NK cells and highly differentiated CD57^+^NKG2A^−^ NK cells are found at higher frequencies in the lungs than in matched peripheral blood. These findings indicate that human lung NK cells have a well-differentiated phenotype (1). Even as early as the human fetal period, the frequency of KIR-expressing and differentiated NK cells is highest in the lungs compared to other tissues (57). A more mature phenotype is also observed for mouse lung NK cells. The most mature subset, CD27^−^CD11b^+^ NK cells, is found at a higher frequency among the lung NK cells (>70%) than those in the liver, peripheral blood, spleen, bone marrow and lymph nodes. Moreover, NK cells in the mouse lung express higher levels of the mature markers CD49b, CD122, CD43, Ly49s, and CD11b, but lower levels of the immature marker CD51, than NK cells in other tissues (56, 58).

Despite the well-differentiated phenotype, both human and mouse lung NK cells are hypofunctional in homeostasis. Human lung NK cells are hyporesponsive to stimulation by target cells (irrespective of priming with IFN-α) compared with peripheral blood NK cells (1). This may be caused by suppressive effects of alveolar macrophages and soluble factors in the epithelial lining fluid of the lower respiratory tract (59). Similarly, in mice, lung NK cells exhibit lower cytotoxicity toward targets compared with spleen NK cells when stimulated by IL-2 or IL-2/IL-12/IL-18 (58). In addition, in mice, the expression intensity of molecules associated with activation (NKp46, NKG2D, and CD69) is lower, and the expression of inhibitory receptors (NKG2A and CD94) is higher, on lung NK cells than on NK cells in the spleen and bone marrow. This indicates that lung NK cells are subject to tighter restrictions in the steady state (56). As the lungs comprise mucosal surfaces that are constantly exposed to environmental and autologous antigens, the dominance of hypofunctional NK cells may contribute to pulmonary homeostasis.

CD49a, CD69, and CD103 are regarded as markers of tissue-resident NK cells (53, 60–63). The fact that the vast majority of lung NK cells in mice are non-tissue-resident cells has been demonstrated by a parabiotic mouse model and the very low expression of CD49a and CD69 (35, 64, 65). Although the majority of human lung NK cells are CD56^dim^ with a non-tissue-resident phenotype (1), a small but distinct CD49a^+^ lung NK cell subset (which largely involves CD56^bright^ NK cells) has recently been identified (66). These CD56^bright^CD49a^+^ lung NK cells strongly co-express CD103 and CD69 and these cells are not found among the CD56^bright^ NK cells in the peripheral blood, implying that CD56^bright^CD49a^+^ lung NK cells may be tissue-resident cells (66). However, the circulating and tissue-resident characteristics of human lung NK cells still need to be further investigated using humanized mice and “multi-omics” analyses (67).

## NK Cells in Lung Infections

There is increasing evidence that NK cells are involved in lung immune responses to respiratory pathogens. As an important type of innate immune cell, NK cells can respond rapidly to invading pathogens and clear them efficiently. On the other hand, NK cells may cause uncontrolled inflammation and pathological damage in some cases.

### Viruses

NK cells are innate immune cells that confer early immunity in acute viral infections and, accordingly, patients with genetic deficiencies that cause the loss of functions of NK cells are subjected to recurrent viral infection (39, 68, 69). However, the rapid immune response mediated by NK cells may sometimes occur at the cost of excessive inflammation. The lungs are continually exposed to various respiratory viruses such as influenza viruses. The evidence of involvement of NK cells in influenza infection dates back to 1982. Ennis et al. (70) demonstrated that individuals infected with influenza viruses exhibit increased peripheral blood NK cell activity in association with interferon (now known as IFN-γ) induction. In mouse models, depletion of systemic or lung NK cells increases the morbidity and mortality of mice during the early course of medium-dose influenza infection (3, 4), indicating a protective role of NK cells. In contrast, depletion of systemic NK cells improves the survival of mice infected with high-dose influenza viruses by alleviating lung immunopathology (5, 6). These findings uncover a dual role for mouse NK cells in influenza infection, providing protection or contributing to pathogenesis, depending on the virus dose.

During influenza infections, NK cells are activated by infected cells via contact-dependent mechanisms (71), and by cytokines such as IL-12, IL-2 and type I IFN, which are derived from infected cells and possibly from other cell types (71–76). In addition to these conventional recognition modes during viral infection, the NK cell receptors NKp44 (which is only expressed on human NK cells) and NKp46 can bind to influenza haemagglutinins (HAs). This allows NK cells to directly recognize influenza viruses and lyse influenza virus-infected cells (77–80). Recent studies have found that influenza vaccines induce the immune memory of human NK cells (81). Similarly, in mice, influenza infection also induces memory-like NK cells, which protect the mice against secondary influenza infection. Intriguingly, these memory-like NK cells reside in the liver rather than in the lungs (82–84), and NK cell-mediated recall responses are not dependent on the NKp46-HA interaction (85).

In mice, NK cells quickly accumulate in the lungs within the first few days of influenza infection (56, 86). These activated lung NK cells then contribute to viral clearance through IFN-γ production, activation of adaptive immune cells, ADCC and cytotoxic lysis. More recently, Kumar et al. (87) reported that conventional NKp46^+^NK1.1^+^CD127^−^RORγt^−^ NK cells in the bronchoalveolar lavage fluid (BALF), trachea and lung tissue produce IL-22 during influenza infection, which facilitates tissue regeneration and prevents excessive lung inflammation. These findings indicate the multiple roles of NK cells in response to influenza viruses.

Due to the difficulties of obtaining lung tissues from humans infected with influenza viruses, most studies exploring the responses of human NK cells to influenza viruses use peripheral blood NK cells (73, 88–90), and less is known about human lung NK cells in influenza infections. Recently, Cooper et al. (66) utilized a lung explant model to characterize human lung NK cells during the early course of influenza infection. The lung NK cells responded quickly upon *ex vivo* influenza infection of lung explants, with upregulation of CD107a by 24 h after infection. Compared with CD56^bright^CD49a^−^ NK cells, CD56^bright^CD49a^+^ lung NK cells, which possibly represent a tissue-resident and trained NK cell subset, express higher levels of CD107a. Recent studies have shown that some activated CD56^dim^CD16^+^ NK cells lose CD16 expression through ADAM17-mediated shedding and become CD56^dim^CD16^−^ NK cells (91). However, the expression of CD107a on CD56^bright^ and CD56^dim^ NK cells is comparable, and there is no difference in expression between CD56^dim^CD16^−^CD49a^+^ and CD56^dim^CD16^−^CD49a^−^ NK cells (66). Although granzyme B and IFN-γ are induced in lung explants after influenza infection, and enhanced IFN-γ responses are detected in peripheral blood NK cells following influenza vaccination (66, 73, 88, 90), there is no direct evidence that granzyme B and IFN-γ are released by lung NK cells. Thus, the immune responses of human lung NK cells in influenza infection remain to be further explored.

Despite the potent antiviral function of NK cells, recurrent influenza infections are common, suggesting that influenza viruses employ complex strategies to evade NK cell-mediated immunosurveillance (92). First, influenza viruses replicate rapidly before NK cells accumulate robustly in the lungs, providing sufficient time for virus dissemination (93). Second, mutation of influenza HA may impair the capacity of NK cells to recognize and lyse infected cells (94). Third, activation of NK cells can be inhibited by influenza HA in a dose-dependent manner (95, 96). On the other hand, when the levels of HA are too low for NK cell recognition, NK cells may not be activated sufficiently to clear viruses (93, 97). Fourth, influenza viruses can directly infect NK cells and induce apoptosis, leading to decreased NK cell cytotoxicity (98).

### Bacteria

NK cells are generally regarded as important contributors to the host defense against tumors and viruses, but recent studies have shown that NK cells also play a role in resisting bacterial infections.

#### Mycobacterium Tuberculosis

Tuberculosis is a leading cause of bacterial infections worldwide. *M. tuberculosis* (MTb) maintains a latent state in most infected individuals, and active disease usually progresses slowly, manifesting later in life (99). *In vitro* studies demonstrate that human peripheral blood NK cells can be activated by MTb-infected monocytes, and this is mediated by NKG2D recognition of ULBP1 and by NKp46 recognition of vimentin (100, 101). Moreover, human NK cells can directly recognize MTb by the binding of TLR2 and NKp44 to peptidoglycan and unknown components of MTb cell walls, respectively, and then become activated (102–104).

A study in immunocompetent mice showed that activated NK cells with upregulated CD69, IFN-γ, and perforin accumulated in the lungs in the early stage after aerosol infection with MTb, but depletion of NK cells did not influence the course of infection (105). Nevertheless, another study in T cell-deficient mice demonstrated that NK cells mediated early defense against MTb infections via IFN-γ (19, 106). Given that mice infected with MTb progress directly to active disease without experiencing latency, these reports indicate the redundant role of NK cells in the active stages of MTb infection. In humans, NK cells in the peripheral blood stimulated with MTb or live *M. bovis* Bacillus Calmette-Guerin (BCG) upregulate IFN-γ expression (107, 108). More recently, Chowdhury et al. (109) conducted a long-term study on a cohort of South African adolescents and found that the frequency of NK cells in the peripheral blood can inform disease progression, therapeutic responses and lung inflammation of patients with active tuberculosis. Pleural fluid, which is the excess fluid that collects around the lungs of pulmonary tuberculosis patients, may be closer to the pulmonary milieus than peripheral blood. The pleural fluid is enriched with IFN-γ-producing CD56^bright^ NK cells due to selective apoptosis of cytotoxic CD56^dim^ NK cells induced by soluble factors present in tuberculous effusions (110). Together, these findings in mice and humans suggest that NK cells may function at the site of active MTb infections mainly through IFN-γ production rather than cytotoxic lysis. Although Chowdhury et al. (109) showed that peripheral blood NK cells from individuals with latent tuberculosis infection display elevated cytotoxicity and increased frequency, whether cytotoxic lysis is employed by NK cells in the defense against MTb, especially latent MTb, remains to be further researched.

#### Klebsiella Pneumoniae

*K. pneumoniae* is an important cause of nosocomial pneumonia and is infamous for multidrug resistance. *In vitro* studies have shown that human NK cells can be activated by TLR2 recognition of recombinant protein A, the pathogen-associated molecular pattern expressed by *K. pneumoniae* (111). However, whether NK cells can recognize live *K. pneumoniae* and lyse *K. pneumoniae* after this direct recognition is unclear. In mice, lung NK cells promote host defense against *K. pneumoniae* by IL-22 and IFN-γ production (13, 14). On the other hand, Wang et al. (112) demonstrated that *K. pneumoniae* pre-infection alleviated influenza virus-induced death and acute lung injury by inhibiting lung NK cell expansion. These findings suggest a complex role of NK cells in response to various pathogens. Thus, accurate and in-depth research into NK cells in different infection conditions is needed and this will contribute to the development of effective interventions for lung infections.

## NK Cells in Lung Inflammation

Asthma and COPD are very common and serious chronic inflammatory diseases of the lungs that may lead to pulmonary fibrosis (113, 114), and NK cells are implicated in both diseases.

### Asthma

Asthma is a chronic airway inflammatory disease, and the majority of cases involve allergic asthma which is typically characterized by type 2 immune responses (114). Asthma can be induced and exacerbated by many factors, such as environmental pollutants, allergens, obesity and viral infections.

Current reports on NK cells in asthmatic patients seem to be somewhat contradictory, both regarding the numbers and functions of NK cells. An early study showed that the percentage of peripheral blood NK cells increases in asthmatic children during acute exacerbations relative to asthmatic children who are in a stable state after prednisolone therapy (115). Nevertheless, Barning et al. (29) and Duvall et al. (116) demonstrated that asthmatic patients have fewer NK cells in both the peripheral blood and BALF than healthy individuals, and the loss of NK cells is increased in patients with severe asthma. The CD56^dim^ NK cell subset (but not the CD56^bright^ NK cell subset) is selectively lost in the peripheral blood of asthmatic patients, whereas BALF NK cells are skewed toward a CD56^dim^ phenotype in asthmatic patients. With regard to the functions of NK cells, there is evidence that NK cells can facilitate inflammation resolution by inducing eosinophil apoptosis (29). In healthy individuals and patients with mild asthma, NK cells in the peripheral blood can induce the apoptosis of eosinophils efficiently. In contrast, despite displaying a more activated phenotype, the cytotoxicity of peripheral blood NK cells from patients with severe asthma is impaired, and the decreased cytotoxicity can be exacerbated by corticosteroids (29, 116). These results indicate the attenuated capacity of NK cells to resolve inflammation in severe asthma. In contrast, earlier studies reported that NK cell cytotoxicity is elevated in the peripheral blood of patients with asthma compared to healthy individuals, and it declines immediately after acute antigen challenge (117, 118). On the other hand, Wei et al. (37) showed that there were increased IL-4^+^ NK cells in the peripheral blood of asthmatic patients compared to healthy individuals, and IL-4^+^ NK cells decreased when the patients recovered owing to erythromycin treatment. This implies a role for NK cells in promoting IgE-mediated ongoing allergic inflammation.

The contradictory results have also been observed in mouse models. The ovalbumin (OVA)-induced asthmatic mouse model is widely used in the study of allergic asthma, and many studies have demonstrated an important role for NK cells at all stages of asthma using this model. OVA sensitization and challenge does not change the total number of NK cells in the lungs, but it selectively increases the number of immature NK cells in the lung draining lymph nodes, as well as upregulating the expression of CD86 on NK cells in both the lungs and lung draining lymph nodes (119). Lack of NK cells either throughout life or just prior to sensitization leads to decreased type 2 cytokine secretion, decreased OVA-specific IgE production, and decreased pulmonary eosinophil infiltration (30, 31). Furthermore, adoptive transfer of OVA-specific T cells from sensitized wild-type (WT) mice, but not mice lacking NK cells, can induce the development of asthma in allergen-challenged RAG^−/−^ mice (31). These results indicate that NK cells are essential for allergic sensitization, and that NK cell-mediated initiation of the type 2 response is probably involved in this process. However, once mice have been sensitized, NK cells may not regulate the established type 2 response but instead they may promote pulmonary eosinophilia, as evidenced by the fact that NK cell depletion during allergen challenge significantly reduces BALF eosinophilia without altering airway hyperresponsiveness or serum OVA-specific IgE levels (119). Nevertheless, Haworth et al. (32) found that depletion of NK cells at the peak of inflammation delays the clearance of airway CD4^+^ T cells and eosinophils. Taken together, these findings suggest that besides the pathologic role of NK cells in allergic sensitization and inflammation promotion, NK cells also provide protection by contributing to the resolution of allergic lung inflammation in mice with OVA-induced asthma. In house dust mite (HDM)-induced asthma mouse models, HDM exposure leads to the accumulation of NK cells in the BALF and lung draining lymph nodes, as well as the activation of NK cells in the lungs (120). Farhadi et al. (33) have shown that NK cells play a critical role in the pathogenesis of HDM-induced asthma via NKG2D and granzyme B. However, a more recent study demonstrated that NK cells are not required for the development of HDM-induced asthmatic disease (120).

There is evidence that viral infection is associated with the development of asthma, and NK cells have been shown to play an important regulatory role in this setting. In mice with pre-existing allergic inflammation and asthma, the induction of asthma-activated NK cells confers more potent protection against influenza infection (121). Nevertheless, NK cells activated by the viral mimic polyinosinic:polycytidylic acid (poly(I:C)) exacerbate OVA-induced asthma via IL-17a production (122). However, when mice are infected with respiratory syncytial viruses and then subjected to allergic sensitization, NK cells inhibit viral- and bystander allergen-specific type 2 responses, possibly through IFN-γ production (7). Recent studies have reported the presence of an altered microbial composition in patients with asthma, and airway dysbiosis is relevant to the clinical features in these individuals (123, 124). Airway colonization by *Haemophilus influenzae* and *Streptococcus pneumonia* at 1 month of age was associated with an increased odds ratio of childhood asthma (125). Although NK cells produced higher levels of IFN-γ during *H. influenzae* and *S. pneumonia* infections (15, 23), colonization by *H. influenzae* and *S. pneumonia* did not inhibit asthma, in contrast to the anti-asthma role of NK cells during respiratory syncytial virus infections. This may be because *H. influenzae* and *S. pneumonia* activate other cell types and pathways involved in asthma occurrence and exacerbations. Moreover, the children were also colonized by many other bacteria, and the integrated effect on NK cells caused by diverse bacteria may lead to variable consequences. Thus, the exact associations between NK cells, airway bacteria and asthma need further study.

The abovementioned contradictory results may be influenced by the fact that asthma-associated factors (such as viral and bacterial infections, obesity, allergens, other environmental insults and corticosteroids) may directly affect NK cell functions and the fact that NK cell-depleting antibodies may impair natural killer T cells (antibodies against NK1.1) or some granulocytes and subsets of T cells in certain conditions (antibodies against asialo-GM1) (126–128). In the future, investigations of the exact roles of NK cells in asthma could be enhanced by using improved tools to specifically deplete lung NK cells, generating transgenic mice that temporarily lack lung NK cells, establishing novel humanized asthmatic mouse models, and carrying out large-scale univariate analyses in asthmatic patients.

## COPD

COPD, caused mainly by cigarette smoking (CS) and biomass fuel, is a common worldwide healthcare issue (129). Chronic inflammation drives the irreversible airway obstruction in COPD, eventually resulting in a decline in lung function (130). Unlike asthma, COPD typically involves the infiltration of neutrophils, Th1 cells and CD8^+^ T cells (130). NK cells are also thought to be responsible for the progression of COPD. Although the number of NK cells in the peripheral blood, BALF and lung parenchyma of COPD patients are the same as in smokers without COPD (34, 131), CD57^+^ cells in pulmonary lymphoid follicles have been reported to be significantly increased in COPD patients compared to in smokers without COPD (132). CS enhances the IL-15 trans-presentation of dendritic cells to induce NK cell priming (133). NK cells exhibit hyperresponsiveness in COPD, as evidenced by the findings that CD16^+^ NK cells kill autologous lung CD326^+^ epithelial cells and that NK cells from CS-exposed mice produce higher levels of IFN-γ upon stimulation with cytokines or TLR ligands (poly(I:C), ssRNA40, or ODN1826) (34, 134, 135). An imbalance of activating and inhibitory signaling contributes to NK cell hyperresponsiveness. NK cells from CS-exposed mice show greater cytotoxic activity in response to the NKG2D ligand RAE-1 (134). Moreover, CS-induced lung inflammation is impaired in NKG2D-deficient mice, revealing the critical role of NKG2D in COPD development (134). In addition, the inhibitory receptor CD94 has been found to be decreased on NK cells of COPD patients, which may be related to increased granzyme B production (131). The state of NK cells in the CS-induced COPD mouse model cannot completely recapitulate that in patients with COPD. BALF NK cells displayed comparable cytotoxic potential between current smokers with COPD and ex-smokers with COPD, suggesting that the alterations of NK cells are not solely caused by CS and that other factors such as genetics and infections may contribute. In contrast, the hyperresponsiveness of NK cells was lost following smoking cessation in the CS-induced COPD mouse model, which indicated the limitation of using this model to study COPD and that a better mouse model is urgently needed.

Bronchial colonization by potentially pathogenic microorganisms is frequently found in COPD, and COPD exacerbations are closely associated with viral and bacterial infections (136–138). An NK cell-related mechanism may contribute to enhanced lung inflammation during influenza-induced COPD exacerbations. In a CS-induced COPD mouse model, after influenza infection, NK cells were found to produce more IFN-γ (135). Microbiome analyses of sputum samples from patients with COPD exacerbations demonstrated an alteration in bacterial diversity, with an overrepresentation of the *Proteobacteria phylum*, which includes most of the bacteria considered to be pathogenic (139). Although chronic colonization by *Pseudomonas aeruginosa* has been found in COPD patients suffering from exacerbations, in most cases, the exacerbations are due to other pathogenic microorganisms (140, 141). Given that lung NK cells in mice infected with *P. aeruginosa* produced increased IFN-γ through NKG2D-mediated activation (17), a phenotype similar to influenza-induced exacerbations, it would be fascinating to investigate the precise effect of *P. aeruginosa-*only infections on COPD exacerbations utilizing murine experiments and to explore the role of NK cell-related NKG2D and IFN-γ in this situation. In addition, colonization by *H. influenzae*, which can lead to NK cell activation (23), has also been reported in COPD patients (142), but the exact interaction between NK cells and *H. influenzae* in COPD is yet to be determined. Collectively, local hyperresponsive NK cells are responsible for smoke-induced lung inflammation, leading to accelerated progression of COPD. Therefore, targeting NK cells may represent a new strategy for treating COPD.

## NK Cells in Lung Cancer

Lung cancer is the leading cause of death related to cancer worldwide (143). It is classified into non-small-cell lung cancer (NSCLC; ~80%) and small-cell lung cancer (SCLC; ~20%). NK cells are cytotoxic lymphocytes that were originally identified based on their ability to kill cancer cells, and their potent antitumor effects have been confirmed in numerous tumor types including lung cancer (144, 145). The localization of NK cells in lung cancer is similar in humans and mice; NK cells are located mostly in the invasive margin surrounding the tumor lesions, with rare direct contact with cancer cells (35, 146, 147).

**Figure 2:**
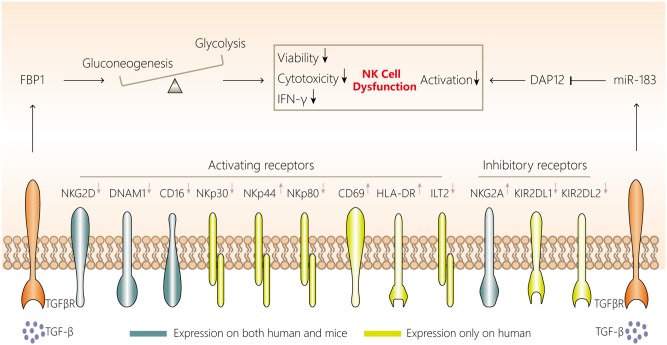
NK cell dysfunction in lung cancer. NK cells in the lung cancer microenvironment display attenuated cytotoxicity, impaired viability and a distinct phenotype, with downregulated expression of NKG2D, DNAM1, CD16, CD27, NKp30, NKp44, and NKp80 and upregulated expression of NKG2A, KIR2DL1, and KIR2DL2. Two mechanisms are involved in NK cell dysfunction in the lung cancer microenvironment. First, aberrant FBP1 expression in NK cells leads to dysfunction by inhibiting their viability and glycolysis. Second, increased microRNA-183 reduces DPA12 expression in NK cells and thus suppresses NK cells. The initiation of both mechanisms may be associated with tumor microenvironment-derived TGF-β. TGF-β, transforming growth factor-β; FBP1, fructose-1,6-bisphosphatase; IFN, interferon; NK, natural killer; DAP12, DNAX activating protein of12 kDa; TGFβR, transforming growth factor β receptor.

The most direct evidence of an anti-lung cancer role for NK cells comes from *Kras*-driven spontaneous lung cancer and cancer cell-injection experiments in mice, in which mice lacking NK cells were generated by *Nfil3* knockout or administration of antibodies against NK1.1 or asialo-GM1. The lung tumor burden was found to be significantly increased in the mice lacking NK cells (35, 36). However, the robust protective role of NK cells against tumors is limited to the early stage of lung cancer, at least in *Kras*-driven lung cancer in mice, because NK cells become dysfunctional during the late stage. In mice, NK cell dysfunction in the lung cancer microenvironment mainly manifests as attenuated cytotoxicity, diminished responsiveness and impaired viability (Figure 2) (35).

Similar phenomena have been observed in NK cells in tumor tissues of patients with NSCLC. NK cells isolated from tumors in these patients have a decreased cell number, a distinctive receptor expression pattern (downregulated expression of NKp30, NKp80, CD16, DNAM1, ILT2, KIR2DL1, and KIR2DL2, but upregulated expression of NKp44, NKG2A, CD69, and HLA-DR), impaired IFN-γ production and CD107a degranulation, lower cytotoxicity, and a proangiogenic phenotype compared with non-tumoral NK cells (146–149). Moreover, NK cells infiltrating NSCLC are enriched with the CD56^bright^CD16^−^ subset (146), which is a minor subset among non-tumoral lung NK cells. The enrichment of CD56^bright^CD16^−^ NK cells is probably due to the exclusion of CD16^+^ NK cells from lung tumor lesions, because the frequency of CD16^−^ NK cells among leukocytes in lung tissues is comparable in tumor and non-tumor tissues (149). However, whether the loss of CD16^+^ NK cells is caused by the impaired viability or failure to infiltrate tumor lesions, and how CD56^bright^CD16^−^ NK cells, which express high levels of the tissue-resident marker CD69 (53), maintain their survival and residency in the lung cancer environment, remain to be determined.

Little is known about NK cells in patients with SCLC. Limited information has shown that, compared to the peripheral blood NK cells of healthy individuals, the peripheral blood NK cells of patients with SCLC are present at the same frequency, exhibit weakened cytotoxicity, and have downregulated NKp46 and perforin expression (150). So far, no studies have reported on the status of NK cells in the tumor microenvironment in patients with SCLC, and this needs to be further investigated.

Platonova et al. (147) found that NK cell infiltration is not correlated with clinical outcomes in NSCLC, similar to the finding by Platonova et al. (147), we recently observed that NK cell depletion in late-stage Kras-driven mouse lung cancer does not influence tumor development (35). The limited prognostic significance of NK cells in NSCLC may be caused by intratumoral NK cell dysfunction in patients who mainly have intermediate- or advanced-stage tumors. Thus, both intratumoral NK cell functions and cell densities may be critical to clinical outcomes in NSCLC. A deeper understanding of the mechanisms associated with NK cell dysfunction in the lung cancer microenvironment will contribute to the development of NK cell-based lung cancer immunotherapy.

NK cell dysfunction in tumor microenvironments can generally be caused by tumor cells, myeloid-derived suppressor cells, macrophages, Tregs and platelets in a contact-dependent manner or via secretion of soluble factors such as transforming growth factor (TGF)-β, IL-10, indoleamine-2,3-dioxygenase, prostaglandin E_2_, and adenosine (145, 151). However, NK cell characteristics may be affected by their tissue localization, and each tumor type has a unique microenvironment composed of diverse immune cells (53). Whether the abovementioned mechanisms are applicable to NK cell dysfunction in lung cancer is yet to be fully investigated. Among these mechanisms, in *Kras*-driven lung cancer in mice, TGF-β may be involved in FBP1 upregulation in NK cells, and FBP1-mediated glycolysis inhibition and FBP1-mediated impaired viability have been confirmed to induce NK cell dysfunction (35). Additionally, Donatelli et al. (152) demonstrated that TGF-β-inducible microRNA-183 silenced human NK cells via DNAX-activating protein of 12 kDa (DAP12) depletion. Moreover, higher levels of TGF-β in the human lung cancer microenvironment and reduced DAP12 expression in tumor-associated NK cells were observed simultaneously, further indicating another TGF-β-involved mechanism associated with NK cell dysfunction.

NK cell dysfunction favors tumor immunoevasion, so focusing on restoring NK cell functions represents important potential strategies for inhibiting lung cancer. These strategies include activating NK cells using IL-2/IL-12/IL-15/IL-18, blocking inhibitory receptors on NK cells by targeting NKG2A, KIR2DL1, and KIR2DL2, enhancing NK cell glycolysis by inhibiting FBP1 and altering the immunosuppressive microenvironment by neutralizing TGF-β.

## Concluding Remarks

Although the biology of NK cells has been well-documented, most studies have focused on peripheral blood NK cells in humans, and bone marrow and spleen NK cells in mice, and less is known about NK cells in the lungs. Recently, the concepts of tissue-resident NK cells and tissue microenvironments have attracted investigators' attention. This has raised the issue that NK cells may be profoundly affected by their local tissue microenvironment, and the characteristics of NK cells in distinct tissues have gradually been uncovered. Our current knowledge about NK cells in the lungs is from studies on WT and transgenic mice and studies comparing healthy individual samples and patient samples (predominately lung tumor resection samples and, less frequently, BALF and sputum samples). The present review shows that NK cells in the lungs appear to be conserved between humans and mice regarding several aspects, including the high degree of differentiation, hypofunction and tissue localization during homeostasis, and responses to tumors and influenza infections. Thus, elaborate mouse models that closely mimic human disease have helped to understand the biology of NK cells in the lungs, such as the *Kras*-driven lung cancer mouse model and influenza viral infection mouse model. However, for some common lung diseases such as tuberculosis, the lack of a good mouse model and the difficulty in obtaining lung tissue from patients had led to a lack of understanding of NK cells in these conditions. Moreover, as mouse NK cells do not express CD56 and KIRs, which are important for human NK cells, the characteristics and functions of different human lung NK cell subsets subdivided by these molecules remain unclear.

Over the past decade, ILCs, which include NK cells, ILC1s, ILC2s, ILC3s, and lymphoid tissue-inducer cells, have emerged as an important cell population with potent roles in host defense in mucosal tissues including lung tissues (153, 154). Generally, ILC1s respond to tumors and intracellular pathogens such as viruses, ILC2s react to extracellular helminths and allergens and ILC3s resist extracellular microbes such as fungi and bacteria; some of these effects have been demonstrated in the lungs (154–158). Although other ILCs in the lungs are far less abundant than NK cells, ILC1s and parts of ILC3s are easily confused with NK cells (43, 44), so previous conclusions about lung NK cells may be influenced by the effects of other ILCs. Thus, it is necessary to exclude other ILCs to further investigate the exact characteristics of lung NK cells.

With regard to lung NK cell research, several interesting issues remain to be solved: (i) whether lung-resident NK cells are present in the lungs in the context of homeostasis and/or disorders; (ii) if so, how lung-resident NK cells function in certain conditions; (iii) the differences and connections between NK cells and ILCs in the lungs; (iv) why memory-like NK cells induced by influenza virus infection are present in the liver rather than in the lungs; (v) whether memory NK cells can form and be maintained in the lungs (as the lungs are frequently challenged by tumors and pathogens); and (vi) how to establish immunocompetent mouse models that can closely mimic human lung diseases. In the future, advanced technologies and tools, such as humanized mice, omics analyses and living microscopy imaging, may be needed to further study lung NK cells. With deeper knowledge of the biology of lung NK cells, effective therapeutic strategies based on NK cells are expected to be applied to treat lung diseases.

## Author Contributions

JC wrote the manuscript. HW designed the review and revised the manuscript.

### Conflict of Interest Statement

The authors declare that the research was conducted in the absence of any commercial or financial relationships that could be construed as a potential conflict of interest.
